# Dual-Beam Steerable TMAs Combining AM and PM Switched Time-Modulation

**DOI:** 10.3390/s22041399

**Published:** 2022-02-11

**Authors:** Roberto Maneiro-Catoira, Julio Brégains, José A. García-Naya, Luis Castedo

**Affiliations:** CITIC Research Center, Department of Computer Engineering, University of A Coruña, Campus de Elviña, 15071 A Coruña, Spain; roberto.maneiro@udc.es (R.M.-C.); julio.bregains@udc.es (J.B.); luis@udc.es (L.C.)

**Keywords:** time-modulated arrays, wireless sensor networks, beam steering, multibeam

## Abstract

Wireless sensor networks (WSN) are increasingly requiring directional antennas that not only provide higher capacity, security, transmission range or robustness against interference, but also contribute with smart antenna capabilities such as adaptive beamforming or multi beam radiation patterns. Standard phased arrays provide these features, but employing feeding networks based on digitally controlled variable phase shifters (VPSs) which have the disadvantage of high cost and limited angular resolution. Instead, time-modulated arrays (TMAs) use switched feeding networks governed by digital periodic sequences which allow harmonic patterns to be radiated and endows (TMAs) with attractive multifunctional capabilities. In this paper, we analyze and properly combine (TMA) switched feeding networks capable of time-modulating an antenna array with discretized amplitude modulation (AM) and phase modulation (PM) waveforms. The advantages of the proposed innovative dual-beam (TMA) with respect to the competing architectures are, on the one hand, its power efficiency and beamsteering (BS) phase sensitivity and, on the other, its hardware simplicity, which allows for an excellent relative cost advantage.

## 1. Introduction

A wireless sensor network (WSN) often consists of spatially distributed autonomous sensors nodes—capable of monitoring in real-time certain physical variables—and one or more base stations connected to the end-users [1]. Major handicaps of WSNs, which typically use omnidirectional antennas, are their need for low energy consumption and a restricted cost of the nodes as well as the guarantee of secure wireless communications within the network.

Contrarily to omnidirectional antennas, directional antennas focus the transmitted power in a narrow radiation region, thus reducing the energy usage and the risk of jammers/eavesdroppers attacks. In addition, by enabling longer transmission distances, they facilitate the use of fewer hops and reduce the risk of any node to become isolated [2]. Hence, the use of directional antennas in WSN allows for improved power and security constraints, but at the expense of a higher complexity [3]. Furthermore, if the nodes are endowed with smart-antenna extra functionalities such as beam steering, direction finding, secure signal transmissions, etc., the antenna feeding network will need to be equipped with variable phase shifters (VPSs), which will substantially increase the costs and radiation power losses. In this regard, the benefits of using directional antennas built with switched beamforming networks (BFNs) rather than VPSs have been investigated [4,5] but in these approaches the utility of the switches consists in shifting between a set of possible positions for different radiation patterns.

A time-modulated array (TMA) is a type of adaptive antenna array which employs switches in the antenna BFN but in a completely different way [6]. The radiation pattern of a TMA is controlled by applying periodical pulses (with fundamental period $T_{0}$) to the individual array elements by means of radio frequency (RF) switches. Such a time-modulation causes the TMA to generate radiation patterns at frequencies $\omega_{c}\pm q\omega_{0}$, where q∈N, $\omega_{c}$ is the carrier frequency, and $\omega_{0}=2\pi /T_{0}$ represents the time-modulation frequency [6]. The attractiveness of using TMAs in WSNs lies mainly in three aspects:

1. The low cost and insertion losses of the array feeding network.
2. The easy and precise method for realising electronic beamsteering by simply controlling the on-off switching instants [7].
3. The utilization of a single RF chain which results in significant savings in power consumption in applications such as simultaneous multi-node communication [8] or channel diversity extraction in multipath wireless reception [9].
4. The ability to transform spatial diversity into frequency diversity which enable smart antenna extra functionalities like direction of arrival (DOA) estimation [10,11,12] or secure communications [13,14], among others.

Despite these advantages, and contrarily to the case of standard phased arrays, the design of TMAs involves dealing with issues that affect the antenna efficiency, namely

1. The difficulty of removing (or strongly attenuate) the unexploited harmonics, including the harmful frequency-mirrored beam patterns. In this sense, the design of the so-called single sideband (SSB) TMAs is of great significance [7,15,16].
2. The presence of signal losses caused by the particular hardware architecture of the TMA switching network [17,18].

Although the design of SSB multibeam TMAs has already been addressed in the literature [18], the corresponding approaches are based on switched BFNs in which amplitude modulation (AM) is exclusively employed. Hence, phase modulation (PM) is not considered. In this work, a fair comparison is made between the two modulation methods when applied to TMAs. Despite that, when considering the same number of discretization levels, AM BFNs allow for a better rejection of unwanted harmonics than PM ones, and the former have the disadvantage of an increased complexity due to the use of power splitters/combiners.

The main contribution of this paper is the design of efficient dual-beam SSB TMAs based on the combination of switched networks, which are simpler than those in [18], capable of generating discretized AM and PM periodic time-modulating signals, and being well-suited for WSN application.

The remainder of the paper is structured as follows. In Section 2, after individually analyzing the spectral characteristics of continuous sinusoidal AM waveforms and continuous linear-phase PM waveforms, we consider the implementation of discretized versions of such signals by means of switched architectures. In Section 3, we combine the previous AM and PM feeding networks to characterize dual-beam steerable TMAs. Section 4 contains numerical simulation results and their discussion. Section 5 compares the proposed dual-beam SSB TMAs with existing dual-beam architectures. Finally, Section 6 is devoted to the conclusions.

## 2. AM and Linear-Phase PM Modulation with Switched Networks

In this section, we start analyzing the original target modulating signals in the analog domain and then we present their corresponding discretized implementations. We study also their characteristics and their implementation with switches.

### 2.1. AM and PM Periodic Modulating Signals

**Figure 1:**
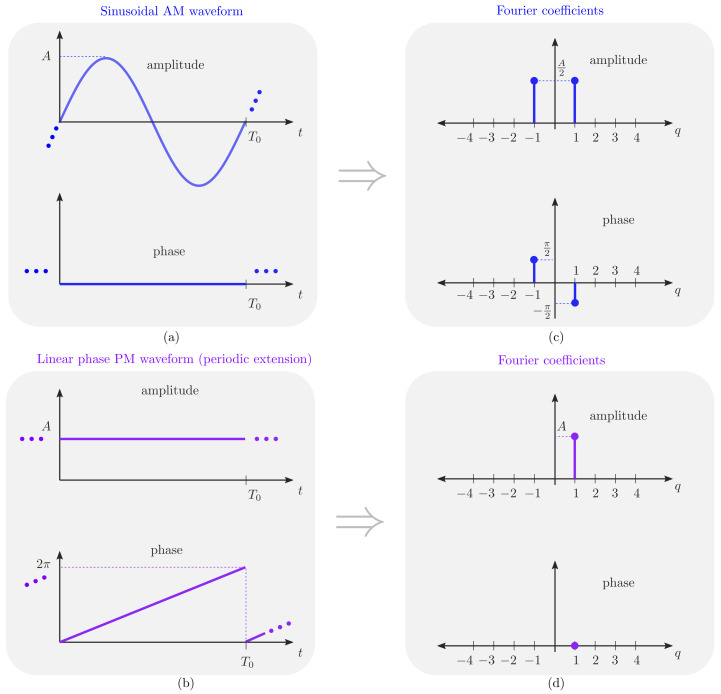
Representation of amplitude and phase versus time. (**a**) A sinusoidal AM waveform with period $T_{0}$. (**b**) The periodic extension of a linear-phase PM waveform. (**c**,**d**) The Fourier coefficients versus *q* of the corresponding signals in (**a**,**b**), respectively (notice that the *q*-th coefficient accounts for the spectral component at the frequency $q2\pi /T_{0}$).

We will begin by interpreting Figure 1 from a TMA perspective. Let us take the simplest case: if we time-modulate each of the the static excitations of a uniform linear array (ULA) with the same periodic signal p(t) with fundamental period $T_{0}$, the radiation patterns appear at frequencies $\omega_{c}\pm q\omega_{0}$ and the *q*-th order Fourier coefficient of p(t) will become the excitation of each element in the corresponding array factor (AF) at the frequency $\omega_{c}+q\omega_{0}$. Hence, intuitively, if we only vary the modulus of the Fourier coefficients (by varying the periodic signals applied to each element), we can modify the topology of the *q*-th power radiated pattern and if we only modify the phases of the coefficients we can shift (i.e., steer) such a power radiated pattern.

When time modulating a ULA with a pure sinusoidal signal (Figure 1a), only two harmonic patterns are generated at the frequencies $\omega_{c}\pm \omega_{0}$ (Figure 1c). The fact that the frequency $\omega_{c}+\omega_{0}$ appears with its mirrored counterpart $\omega_{c}-\omega_{0}$ means that we have a double sideband (DSB) TMA, which generates harmonic diagrams with the same shape (Fourier coefficients with the same modulus), but pointing towards directions that are symmetric (opposite phase) with respect to the broadside of the array. In other words, both harmonics do not have independent phases and it is not possible to steer them independently, thus loosing flexibility and energy efficiency. Hence, although in this work we will profitably exploit the DSB feature of discretized sinusoids to perform independent dual-beamsteering (as we will see in the ensuing section), TMA research generally focuses on the synthesis of SSB TMAs without frequency-mirrored beampatterns.

In this sense, if we consider the periodic extension of a linear-phase PM waveform (Figure 1b), we observe that it has a single nonzero Fourier coefficient at q=1 (see Figure 1d). Therefore, if we apply such a periodic pulse to time-modulate the elements of a ULA, we will obtain a single harmonic pattern at $\omega_{c}+\omega_{0}$, thus achieving SSB TMA capabilities.

Finally, notice that if $P_{q}$ are the Fourier coefficients of a periodic signal p(t), then $e^{-j\omega_{0}q\tau }P_{q}$ are the Fourier coefficients of p(t−τ). Consequently, if p(t) (which could represent the signal in Figure 1a or in Figure 1b) is time-shifted, then the phases of its Fourier coefficients are modified proportionally to the time delay τ and, accordingly, the *q*-th harmonic pattern can be steered to a given direction.

### 2.2. Discretization of the Modulating Signals: Characteristics and Generation

**Figure 2:**
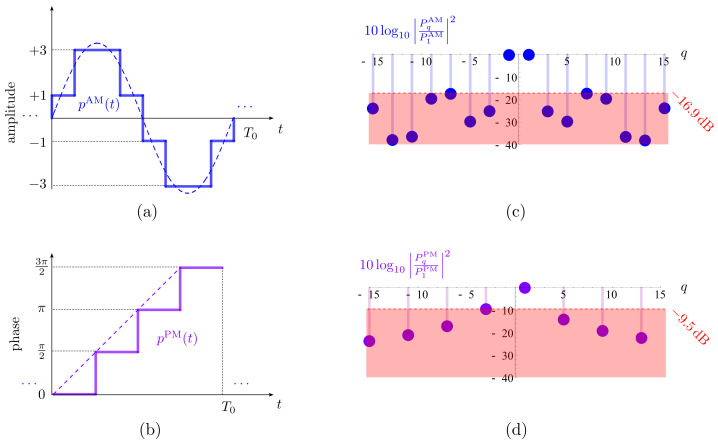
Four-level discretization of the waveforms shown in Figure 1: (**a**) discretized sinusoidal AM waveform (one period); (**b**) Discretized linear-phase PM waveform (one period). Normalized Fourier series power spectrum of the discretized waveforms: (**c**) AM waveform in (**a**); (**d**) PM waveform in (**b**).

In this work, we will consider two types of waveform discretization. One is shown in Figure 2 and uses only four different levels. Such a discretization has the particularity of being easily implementable by means of switch architectures. Figure 2a shows a four-level discretized version of a sinusoidal AM waveform denoted by $p^{\mathrm{AM}}\left(t\right)$. We can observe in Figure 2c its normalized Fourier series power spectrum in dB, $20\log_{10}\left|P_{q}^{\mathrm{AM}}/P_{1}^{\mathrm{AM}}\right|$, being $P_{q}^{\mathrm{AM}}$ the Fourier coefficient of order *q*. We can see, apart from the DSB character of the waveform, that $p^{\mathrm{AM}}\left(t\right)$ is a very good approximation of the analog sine function because, although there are nonzero harmonics other than the useful ones (q±1), they are all attenuated by at least 16.9 dB with respect to the useful harmonics. More specifically, $\Psi^{\mathrm{AM}}=\left\{q=2k+1;k\in Z\right\}=\left\{\ldots ,-5,-3,-1,1,3,5,\ldots \right\}$ is the set of indexes of the nonzero harmonics.

**Figure 3:**
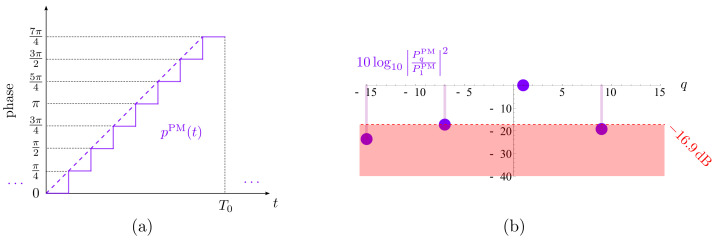
(**a**) Discretized linear-phase PM waveform (one period) with 8 levels. (**b**) Normalized Fourier series power spectrum of the discretized waveform.

Figure 2b shows a 4-level discretization of the periodical extension of a linear-phase PM waveform denoted $p^{\mathrm{PM}}\left(t\right)$ and Figure 2d shows its corresponding normalized Fourier series power spectrum. Observe the SSB characteristic of the waveform while the most meaningful undesirable harmonic is the one with order q=−3 whose relative level is −9.5 dB with respect to the useful harmonic q=1. Note that for this level of discretization, only harmonics whose order *q* belongs to the set $\Psi^{\mathrm{PM}}=\left\{q=4\Gamma -3;\Gamma \in Z\right\}=\left\{\ldots ,-7,-3,1,5,9,13,\ldots \right\}$ are generated. The rejection level of unwanted harmonics can be improved by considering eight discretization levels, see Figure 3. It can be seen that in this case a minimum rejection of unwanted harmonics of at least −16.9 dB is achieved, being $\Psi^{\mathrm{PM}}=\left\{q=8\Lambda -23;\Lambda \in Z\right\}=\left\{\ldots ,-15,-7,1,9,17,25,\ldots \right\}$ the set of indexes of the non-zero harmonics. Ultimately, to obtain the same level of rejection of unwanted harmonics, it is necessary to discretize the PM signal with twice as many levels as the AM signal.

**Figure 4:**
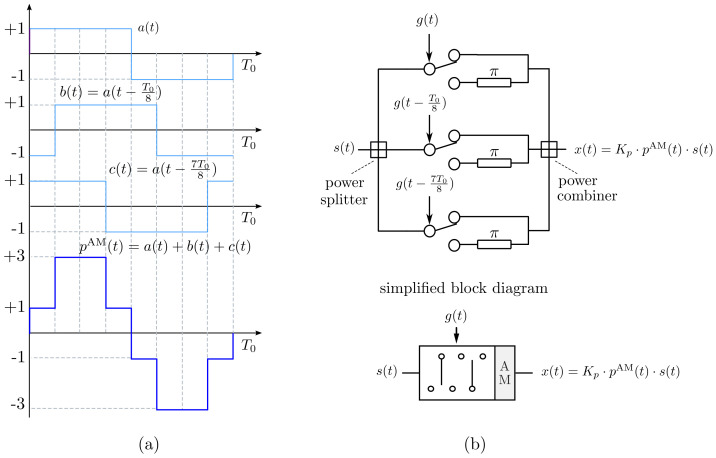
(**a**) Implementation of the periodic AM modulating pulse $p^{\mathrm{AM}}\left(t\right)$ from three periodic bipolar square pulses shifted in time. (**b**) SPDT architecture capable of time modulating an input signal s(t) with $p^{\mathrm{AM}}\left(t\right)$ (top) and its simplified block diagram (bottom).

Let us now focus on the design of the switching networks capable of implementing the discretized signals in Figure 2, starting with the discretized sinusoidal AM waveform $p^{\mathrm{AM}}\left(t\right)$ in Figure 2a. Such a waveform can be constructed as the sum of three periodic bipolar square pulses that have a certain fixed time delay between them [18]. Time modulating with $p^{\mathrm{AM}}\left(t\right)$ is efficiently implemented with the single-pole dual-throw (SPDT) switches connection shown in Figure 4b where the switches are controlled by delayed versions of the same unipolar squared pulse g(t). Notice that the unipolar signal that controls the switches is g(t)=1 if a(t)≥0, and g(t)=0 if a(t)<0. At the output of this SPDT switched feeding network we have the signal $x\left(t\right)=k_{p}p\left(t\right)s\left(t\right)$, with s(t) being the input signal and $k_{p}^{2}=1/5$ a normalizing constant resulting from matching the powers of s(t) and x(t) because ideal SPDT switches do not waste power since they have no off-state [18]. For the sake of clarity, the feeding network of Figure 4b (top) is simplified by means of the block diagram labeled “AM” in Figure 4b (bottom) and will be one of the two blocks involved in the synthesis of the dual-beam steerable TMA considered in Section 3.

**Figure 5:**
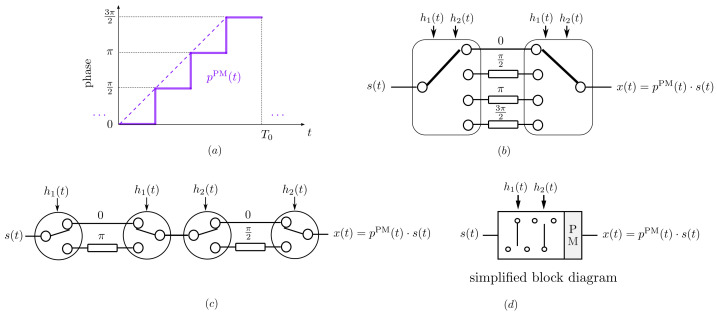
(**a**) 4-level linear phase periodic pulsed waveform $p^{\mathrm{PM}}\left(t\right)$. (**b**) SP4T architecture capable of time modulating an input signal s(t) with $p^{\mathrm{PM}}\left(t\right)$. (**c**) SPDT architecture capable of time modulating an input signal s(t) with $p^{\mathrm{PM}}\left(t\right)$. Note that none of the architectures (**b**,**c**) require power splitters/combiners. (**d**) Equivalent block diagram.

The design of a switched network capable of applying the periodical extension of a 4-level linear-phase PM waveform (see Figure 5a) can be realized either with two single-pole four-throw (SP4T) switches (Figure 5b) or with four SPDT switches (Figure 5c). In both cases, the unipolar signals that control the switches, $h_{1}\left(t\right)$ and $h_{2}\left(t\right)$, and the phase shifts applied to the input signal, $p^{\mathrm{PM}}\left(t\right)$, i.e., the phases of $∢p^{\mathrm{PM}}\left(t\right)$, are the following:

- $h_{1}\left(t\right)=0$, $h_{2}\left(t\right)=0$→$∢p^{\mathrm{PM}}\left(t\right)=0$
- $h_{1}\left(t\right)=0$, $h_{2}\left(t\right)=1$→$∢p^{\mathrm{PM}}\left(t\right)=\pi /2$
- $h_{1}\left(t\right)=1$, $h_{2}\left(t\right)=0$→$∢p^{\mathrm{PM}}\left(t\right)=\pi$
- $h_{1}\left(t\right)=1$, $h_{2}\left(t\right)=1$→$∢p^{\mathrm{PM}}\left(t\right)=3\pi /2$

Analogously to the AM case, the PM feeding network is abstracted in the block diagram labeled “PM” (see Figure 5d). Notice that for the case of an 8-level linear-phase PM waveform (see Figure 3a) the corresponding switched network can be realized either with two single-pole eight-throw (SP8T) switches or with eight SPDT switches. In both cases, three unipolar control sequences, $h_{1}\left(t\right)$, $h_{2}\left(t\right)$ and $h_{3}\left(t\right)$ will be required.

## 3. Design of Dual-Beam Steerable TMAs Combining AM and PM BFNs

**Figure 6:**
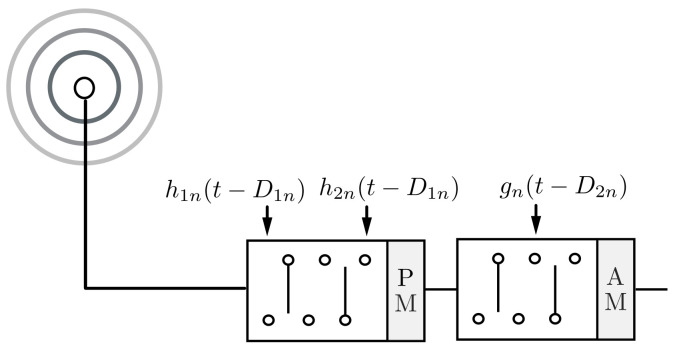
Block diagram of the *n*th element feeding network of the TMA approach that allows the independent steering of two harmonic beam patterns by combining AM and PM time-modulation.

The AM and PM switched modules detailed above are used as the BFN building blocks of a receive dual-beam steerable TMA approach (see a single antenna element in Figure 6). Without loss of generality, in the subsequent analysis we consider the case of a 4-level linear-phase PM switched network. In what follows, we will explain how this feeding network is able to independently steer two antenna array beams by properly selecting the time-delay parameters $D_{1n}$ and $D_{2n}$ shown in Figure 6.

The array factor (AF) of a TMA (as in any antenna array) represents its response for the case of isotropic elements. The hallmark of the TMA technique is that the array excitations are time-modulated and, as a result, the AF of a TMA is time-variant. Accordingly, by considering a linear TMA with time modulated excitations $u_{n}\left(t\right)$, n∈{0,…N−1}, the time-varying AF (explicitly including the term $e^{j\omega_{c}t}$) is given by

(1) $$\begin{array}{cc} F(\theta ,t) & =e^{j\omega_{c}t}\sum_{n=0}^{N-1}u_{n}\left(t\right)e^{j\beta_{c}z_{n}\cos\theta } \end{array}$$

where $z_{n}$ represents the position of the *n*-th array ideal (i.e., perfectly conductive) isotropic element on the *z* axis, θ is the angle with respect to such a main axis, and $\beta_{c}=2\pi /\lambda_{c}$ is the wavenumber for a carrier wavelength $\lambda_{c}=2\pi c/\omega_{c}$, being $\omega_{c}$ the carrier frequency.

According to Figure 6, the time modulated excitations are

(2) $$\begin{array}{cc} v_{n}\left(t\right) & =p^{\mathrm{PM}}\left(t-D_{1n}\right)\cdot K_{p}p^{\mathrm{AM}}\left(t-D_{2n}\right) \end{array}$$

As described below, the time delays $D_{1n}$ and $D_{2n}$ can be selected to accomplish the directions of the main lobes of the two independently-steerable beams ($\theta_{a}$ and $\theta_{b}$, respectively) in the proposed TMA approach.

By considering the Fourier series expansion of $p^{\mathrm{AM}}\left(t\right)$ and $p^{\mathrm{PM}}\left(t\right)$ together with the time-shifting property of the Fourier coefficients in Equation (2), we can readily obtain

(3) $$v_{n}\left(t\right)=K_{p}\sum_{q\in \Psi^{\mathrm{PM}}}P_{q}^{PM}e^{-jq\omega_{0}D_{1n}}e^{jq\omega_{0}t}\cdot \sum_{i\in \Psi^{\mathrm{AM}}}P_{i}^{AM}e^{-ji\omega_{0}D_{2n}}e^{ji\omega_{0}t}$$

To simplify the notation, we now define the dynamic excitations as

(4) $${}_{i}^{q}I_{n}=k_{p}P_{q}^{PM}P_{i}^{AM}e^{-j\omega_{0}\left(qD_{1n}+iD_{2n}\right)}$$

Which allows for reducing Equation (3) to

(5) $$v_{n}\left(t\right)=\sum_{q\in \Psi^{\mathrm{PM}}}\sum_{i\in \Psi^{\mathrm{AM}}}{}_{i}^{q}I_{n}e^{j\left(q+i\right)\omega_{0}t}.$$

Finally, substituting Equation (5) into Equation (1), we arrive at the time-variant AF

(6) $$\begin{array}{cc} F(\theta ,t) & =\sum_{q\in \Psi^{\mathrm{PM}}}\sum_{i\in \Psi^{\mathrm{AM}}}\sum_{n=0}^{N-1}{}_{i}^{q}I_{n}e^{j\beta_{c}z_{n}\cos\theta }e^{j(\omega_{c}+\left(q+i\right)\omega_{0})t}=\\ & =\sum_{q\in \Psi^{\mathrm{PM}}}\sum_{i\in \Psi^{\mathrm{AM}}}F_{i}^{q}\left(\theta \right)e^{j(\omega_{c}+\left(q+i\right)\omega_{0})t} \end{array}$$

where

(7) $$F_{i}^{q}\left(\theta \right)=\sum_{n=0}^{N-1}{}_{i}^{q}I_{n}e^{j\beta_{c}z_{n}\cos\theta }$$

is the spatial AF at the frequency $\omega_{c}+\left(q+i\right)\omega_{0}$.

In view of Equation (4) and the harmonics profile in Figure 2c,d, we observe that the most meaningful harmonic patterns in Equation (6), in decreasing order of significance, are the ones shown in Table 1. In such a table, we consider the relative level of the maximum of a given harmonic pattern $\left|F_{i}^{q}\left(\theta_{\max}\right)\right|$ with respect to the maximum of $\left|F_{1}^{1}\left(\theta_{\max}\right)\right|$ (which we take as a reference pattern) by defining ${}_{i}^{q}\Delta_{\mathrm{peak}}=20\log\left|F_{i}^{q}\left(\theta_{\max}\right)/F_{1}^{1}\left(\theta_{\max}\right)\right|$, being $\theta_{\max}$ the direction in which an AF amplitude is maximum. According to Table 1, the useful harmonics are $\omega_{c}+2\omega_{0}$ (i.e., *q* = *i* = 1) and $\omega_{c}$ (i.e., *q* = 1 and *i* = −1) because their beam patterns, $F_{-1}^{1}\left(\theta \right)$ and $F_{1}^{1}\left(\theta \right)$, have a peak level 9.5 dB above the remaining ones, i.e., the two beam patterns $F_{-1}^{1}\left(\theta \right)$ and $F_{1}^{1}\left(\theta \right)$ are the ones to be used to perform beam-steering.

We now assume that the array inter-element distance is $\lambda_{c}/2$. Hence, $\beta_{c}z_{n}=\pi n$ and, according to Equation (7) and Equation (4), the spatial AFs of the useful harmonics simplify to

(8) $$\begin{array}{cc} F_{1}^{1}\left(\theta \right) & =\sum_{n=0}^{N-1}k_{p}P_{1}^{\mathrm{PM}}P_{1}^{\mathrm{AM}}\cdot e^{j(n\pi \cos\theta -\omega_{0}\left(D_{1n}+D_{2n}\right))},\\ F_{-1}^{1}\left(\theta \right) & =\sum_{n=0}^{N-1}k_{p}P_{1}^{\mathrm{PM}}P_{-1}^{\mathrm{AM}}\cdot e^{j(n\pi \cos\theta -\omega_{0}\left(D_{1n}-D_{2n}\right))} \end{array}$$

Therefore, if we are willing to steer these beampatterns towards the directions $\theta_{a}$ and $\theta_{b}$, the following two conditions must, respectively, hold

(9) $$\begin{array}{cc} n\pi \cos\theta_{a} & =\omega_{0}\left(D_{1n}+D_{2n}\right),\\ n\pi \cos\theta_{b} & =\omega_{0}\left(D_{1n}-D_{2n}\right) \end{array}$$

which lead to the following time delays

(10) $$\begin{array}{cc} D_{1n} & =\frac{nT_{0}\left(\cos\theta_{a}+\cos\theta_{b}\right)}{4}\\ D_{2n} & =\frac{nT_{0}\left(\cos\theta_{a}-\cos\theta_{b}\right)}{4}. \end{array}$$

In case of using an 8-level discretization in the PM feeding network, we obtain similar results, but with a higher level of rejection of the undesired harmonics, being the ${}_{i}^{q}\Delta_{\mathrm{peak}}$ values of the most meaningful unwanted harmonics equal to −16.9 dB, as shown in Table 2.

## 4. Numerical Simulations

An insightful figure of merit to be considered by a TMA designer is the so-called time-modulation radiation efficiency [9] $\eta_{\mathrm{TM}}=P_{U}^{\mathrm{TM}}/P_{R}^{\mathrm{TM}}$, where $P_{U}^{\mathrm{TM}}$ and $P_{R}^{\mathrm{TM}}$ are the useful and total average power values radiated by the TMA, respectively. Notice that $\eta_{\mathrm{TM}}$ accounts for the ability of a TMA to filter out and radiate only over the useful harmonics. By considering $z_{n}=n\lambda_{c}/2$ in Equation (1), according to [9,18], we can calculate such an efficiency by means of Equation (4):

(11) $$\eta_{\mathrm{TM}}=\frac{P_{U}^{\mathrm{TM}}}{P_{R}^{\mathrm{TM}}}=\frac{\sum_{n=0}^{N-1}{\left|{}_{1}^{1}I_{n}\right|}^{2}+\sum_{n=0}^{N-1}{\left|{}_{-1}^{1}I_{n}\right|}^{2}}{\sum_{n=0}^{N-1}\sum_{q\in \Psi^{\mathrm{PM}}}\sum_{i\in \Psi^{\mathrm{AM}}}{\left|{}_{i}^{q}I_{n}\right|}^{2}}$$

For the case of a configuration with 4-level AM and 4-level PM waveforms, the radiation efficiency is $\eta_{\mathrm{TM}}$ = 0.7654 whereas with 4-level AM and 8-level PM waveforms is $\eta_{\mathrm{TM}}$ = 0.8974.

**Figure 7:**
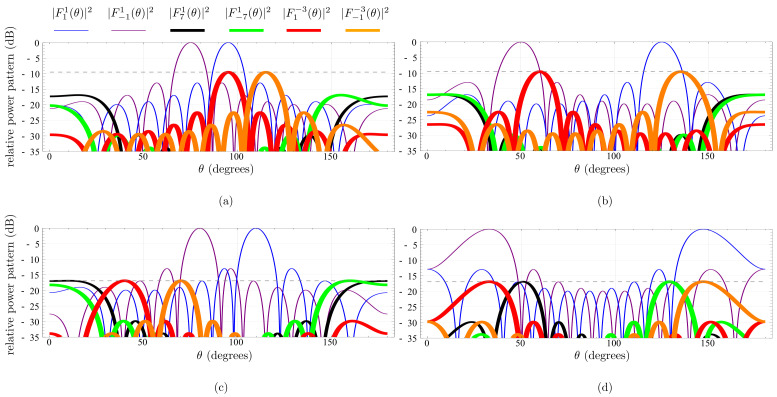
Relative power patterns (dB) for N=10. (**a**) Dual-beam steerable TMA approach in Figure 6, i.e., considering 4-level AM and 4-level PM waveforms where the two useful beam patterns are pointing towards the directions $75^{\circ }$ and $95^{\circ }$, respectively. (**b**) The same TMA approach as in (**a**), but the pointing directions are $50^{\circ }$ and $125^{\circ }$. (**c**) Dual-beam steerable TMA approach considering 4-level AM and 8-level PM waveforms where the two useful beam patterns are pointing towards the direction of $80^{\circ }$ and $110^{\circ }$. (**d**) The same TMA approach as in (**c**), but considering a scenario where the two useful beam patterns point towards $33^{\circ }$ and $147^{\circ }$ (i.e., $\pm 57^{\circ }$ from the broadside direction) which correspond to the limits of the scanning interval that each useful beam pattern is able to support.

We next consider $f_{c}=\omega_{c}/\left(2\pi \right)=2.4$ GHz, $f_{0}=\omega_{0}/\left(2\pi \right)=5.5$ MHz, *N* = 10 and the four scenarios shown in Figure 7a,b with 4-level AM and 4-level PM modulating waveforms, and (c,d) with 4-level AM and 8-level PM. Different steering directions, $\theta_{a}$ and $\theta_{b}$, are considered along the scenarios for the exploited harmonics and, accordingly, the normalized time delays $D_{1n}$ and $D_{2n}$ are set after Equation (10). We validate that the dual-beam steerable TMA approach is able to concentrate the radiated power (recall that $\eta_{\mathrm{TM}}=76.5\%$ and $\eta_{\mathrm{TM}}=89.8\%$, respectively) on the desired harmonic patterns, $|F_{1}^{1}{\left(\theta \right)|}^{2}$ and $|F_{-1}^{1}{\left(\theta \right)|}^{2}$, located at $\omega_{c}$ and $\omega_{c}+2\omega_{0}$, respectively. Hence, simulations show the beam steering and harmonic rejection capabilities of the approach for the two types of discretization of the PM waveform (−9.5 dB and −16.9 dB respectively). The characteristics of the dominant harmonic patterns are the ones described in Table 1 and Table 2 and plotted with a thick line in Figure 7. The limits of the scanning interval that each useful beam pattern is able to support are $33^{\circ }$ and $147^{\circ }$. Within these limits the maximum level of undesirable harmonics remain below the threshold indicated in Table 1 and Table 2, respectively. The Half Power Beamwidth (HPBW) of the useful harmonics radiation patterns increases slightly as the steering direction separates from the broadside direction.

## 5. Comparison with Existing Dual-Beam Architectures

In this section we compare the proposed dual-beam steerable TMA that combines AM and PM switched modulation (Figure 6) with the following equivalent architectures: (1) standard dual-beam phased arrays employing digital VPS, and (2) dual-beam steerable TMAs capable of offering features similar to the previous ones. To make a fair comparison, existing TMA approaches must be able to do a flexible beamsteering (BS) of the two beams. In the literature about TMAs, the vast majority of papers present a deficient dual-beam capability in the sense that TMAs cannot perform an arbitrary and independent two-beam BS due to the linear dependence between the harmonic excitation phases (see, e.g., [9,13]), thus showing a significant inflexibility. Furthermore, such dual-beam TMAs do not remove the corresponding negative harmonic radiated patterns (or mirrored patterns), i.e., they have no SSB features, being sensibly more inefficient. Hence, within the scope of the existing TMAs, a fair comparison is restricted to dual-beam steerable SSB TMAs [18].

In order to consider the hardware insertion losses in the antenna feeding networks, we must set a suitable framework for comparison. Accordingly, we focus our analysis on the typical WSN band ($f_{c}=2.4$ GHz) supporting channels with a bandwidth B=5 MHz. Subsequently, we consider a TMA frequency $f_{0}=5.5$ MHz, and $1/t_{\mathrm{SW}}^{\mathrm{speed}}=20$ MHz (maximum speed supported by the switches, with $t_{\mathrm{SW}}^{\mathrm{speed}}=t_{\mathrm{ON}}+t_{\mathrm{OFF}}=50$ ns [18,19]) in order to ensure that the inequality $B<f_{0}<1/t_{\mathrm{SW}}^{\mathrm{speed}}$ [18] is fulfilled. We also select for comparison the less complex version of the proposed design, i.e., the feeding network in Figure 6, in which the corresponding PM block is governed by a 4-level linear phase waveform, and choosing the hardware with SPDT switches shown in Figure 5c.

**Figure 8:**
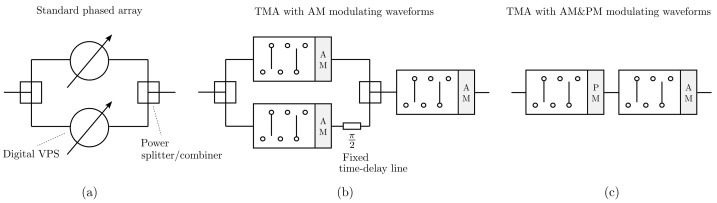
Block diagrams of the corresponding feeding networks of each antenna for different dual-beam steerable array solutions: (**a**) Standard phased array with digital VPSs [26]; (**b**) TMA with AM modulating waveforms [18]; and (**c**) TMA with AM & PM modulating waveforms (this work).

Table 3 shows the hardware devices considered for the design of the antenna feeding networks in Figure 8. Notice that TMA techniques provide beam steering with an excellent phase sensitivity since the phases of the antenna excitations can be adjusted continuously [7,20] by simply modifying the switch-on time instants of the periodic sequences controlling the switches. Hence, in order to make a fair comparison with standard phase arrays, we consider—as in [18]—high resolution (6 bits) digitally controlled phase shifters [21,22,23] (see Table 3) leading to phase steps of $\theta_{\mathrm{step}}=360^{\circ }/2^{6}=5.6^{\circ }$. The comparison between the architectures illustrated in Figure 8 focuses on several decisive aspects in WSN: power efficiency, phase sensitivity, complexity, and cost.

**Table 3:** Examples of devices working in the $f_{c}=2.4$ GHz WSN frequency band that can be used in a suitable implementation of the dual-beam steerable arrays in Figure 8.

Device	Frequency (GHz)	Insertion Loss (dB)	References
6-bit VPS	S band	$\eta_{\mathrm{VPS}}=4.64$	[21,22,23]
2-way splitter/combiner	1.7–3 (S band)	$\eta_{2-\mathrm{way}}=$ 0.5	[24]
3-way splitter/combiner	1.6–2.8 (S band)	$\eta_{3-\mathrm{way}}=$ 0.8	[24]
SPDT RF switch	0.05–26.5	$\eta_{\mathrm{SPDT}}=$ 0.4	[23]
time delay-line (PCB)	2.4 ± 0.0025	$\eta_{\mathrm{delay}-\mathrm{line}}<$ 0.06	[25]

- Power efficiency takes into account the insertion losses associated with the specific hardware devices employed, $\eta_{\mathrm{HW}}$, and additionally, in the case of the TMA technology, it also considers the time-modulation radiation efficiency, $\eta_{\mathrm{TM}}$ Equation (11), due to the fact that non-useful harmonics are also radiated, hence wasting power.
  In view of Figure 8a, the power efficiency of the dual-beam standard phased array feeding network is
  (12) $$\begin{array}{c} \eta_{\mathrm{PA}(\mathrm{dB})}=\eta_{\mathrm{HW}(\mathrm{dB})}^{\mathrm{PA}}=2\eta_{2-\mathrm{way}(\mathrm{dB})}+\eta_{\mathrm{VPS}(\mathrm{dB})}=5.64\;\mathrm{dB} \end{array}$$
  For the case of the TMA architecture in Figure 8b, which was thoroughly analyzed in [18], the power efficiency obeys to
  (13) $$\eta_{\mathrm{TMA}(\mathrm{dB})}^{\mathrm{AM}}=\eta_{\mathrm{TM}(\mathrm{dB})}^{\mathrm{AM}}+\eta_{\mathrm{HW}(\mathrm{dB})}^{\mathrm{AM}}$$
  with $\eta_{\mathrm{TM}(\mathrm{dB})}^{\mathrm{AM}}=0.49$ dB, whereas (see Figure 8b):
  (14) $$\eta_{\mathrm{HW}(\mathrm{dB})}^{\mathrm{AM}}=2\eta_{\mathrm{AM}(\mathrm{dB})}^{\mathrm{module}}+2\eta_{2-\mathrm{way}(\mathrm{dB})}+\eta_{\mathrm{delay}-\mathrm{line}(\mathrm{dB})}$$
  being
  (15) $$\eta_{\mathrm{AM}(\mathrm{dB})}^{\mathrm{module}}=2\eta_{3-\mathrm{way}(\mathrm{dB})}+\eta_{\mathrm{SPDT}(\mathrm{dB})}+\eta_{\mathrm{delay}-\mathrm{line}(\mathrm{dB})}$$
  which, by substituting successively the values from Table 3 in Equations (13)–(15), leads to
  (16) $$\eta_{\mathrm{TMA}(\mathrm{dB})}^{\mathrm{AM}}=5.67\;\mathrm{dB}$$
  Regarding the antenna feeding network proposed in this article (see Figure 8c), we have
  (17) $$\eta_{\mathrm{HW}(\mathrm{dB})}^{\mathrm{AM}/\mathrm{PM}}=\eta_{\mathrm{AM}(\mathrm{dB})}^{\mathrm{module}}+\eta_{\mathrm{PM}(\mathrm{dB})}^{\mathrm{module}}$$
  where
  (18) $$\eta_{\mathrm{PM}(\mathrm{dB})}^{\mathrm{module}}=4\eta_{\mathrm{SPDT}(\mathrm{dB})}+2\eta_{\mathrm{delay}-\mathrm{line}(\mathrm{dB})}$$
  and since $\eta_{\mathrm{TM}(\mathrm{dB})}^{\mathrm{AM}/\mathrm{PM}}=-10\log_{10}\left(0.7654\right)=1.16$ dB, we have that (Table 3)
  (19) $$\eta_{\mathrm{TMA}(\mathrm{dB})}^{\mathrm{AM}/\mathrm{PM}}=\eta_{\mathrm{TM}(\mathrm{dB})}^{\mathrm{AM}/\mathrm{PM}}+\eta_{\mathrm{HW}(\mathrm{dB})}^{\mathrm{AM}/\mathrm{PM}}=4.94\;\mathrm{dB}$$
  Note that the proposed antenna feeding network offers the best figure in terms of power efficiency.
- Phase sensitivity. We compare the minimum phase step, $\theta_{\mathrm{step}}$, that each steering array architecture is capable of providing. We saw at the beginning of this section that, in the case of a standard phased array (see Figure 8a) with 6-bit VPSs, $\theta_{\mathrm{step}}=5.6^{\circ }$. The great advantage of TMA architectures (see Figure 8b,c) is that $\theta_{\mathrm{step}}$ is directly proportional to $t_{\mathrm{SW}}^{\mathrm{speed}}$ [18]. By considering the SPDT switches in Table 3, with $t_{\mathrm{SW}}^{\mathrm{speed}}=50$ ns, we achieve (analogously to [18]) a $\theta_{\mathrm{step}}=1.7^{\circ }$, which is significantly better than that offered by a standard architecture with 6-bit VPSs.
- The hardware complexity analysis is necessarily centered on two elements: the number of SPDT switches and the number of power splitter/combiners employed by the BFN architecture. Note that a *b*-bit digital phase shifter usually employs 2b SPDT switches [21,22,23]. Thus, the BFN of a standard dual-beam phased array with 6-bit VPSs (see Figure 8a) will have 24 SPDT switches. On the other hand, the dual-beam TMA with AM waveforms shown in Figure 8b requires 9 SPDT switches, whereas the proposed TMA combining AM with PM waveforms (see Figure 8c) uses only 7 SPDT switches. If we now compare the number of power splitter/combiners required by the three architectures, we observe that the TMA architecture with AM waveforms quadruplicates the number of these elements with respect to the other two (i.e., 8 versus 2). Thus, it is concluded that the proposed architecture is objectively the most advantageous in terms of complexity.
- Cost effectiveness. Among all the hardware devices listed in Table 3, the most expensive is clearly the 6-bit VPSs. Therefore, we will analyze the percentage of savings of each TMA architecture with respect to the standard phased array. We can see that the strongest differential aspect of the proposed TMA architecture for WSN applications (see Figure 8c) is the significant cost savings. All quantitative results of the comparative are presented in Table 4.

## 6. Conclusions and Future Work

An innovative combination of efficient TMA switched feeding networks capable of time-modulating an antenna array with discretized AM and PM waveforms has been proposed. Such architectures are attractive for WSN applications since they allow high resolution dual-beam steering antenna arrays at a reduced complexity and competitive cost.

Although the results of this analysis are promising, we are aware that there are future lines of research that remain open. Next steps are the realization of full-wave simulations, and the construction and characterization of a hardware prototype.

## Figures and Tables

**Table 1:** Characteristics of the dominant harmonic patterns of Equation (6) in decreasing order of significance. Both AM and PM modulating waveforms are 4-level. The useful harmonics are shaded in light blue.

*q*	*i*	Frequency	Dynamic Excitations (${}_{i}^{q}I_{n}$)	${}_{i}^{q}\Delta_{\mathrm{Peak}}$ (dB)
1	1	$\omega_{c}+2\omega_{0}$	$k_{p}P_{1}^{\mathrm{PM}}P_{1}^{\mathrm{AM}}\cdot e^{-j\omega_{0}\left(D_{1n}+D_{2n}\right)}$	0
1	−1	$\omega_{c}$	$k_{p}P_{1}^{\mathrm{PM}}P_{-1}^{\mathrm{AM}}\cdot e^{-j\omega_{0}\left(D_{1n}-D_{2n}\right)}$	0
−3	1	$\omega_{c}-2\omega_{0}$	$k_{p}P_{-3}^{\mathrm{PM}}P_{1}^{\mathrm{AM}}\cdot e^{-j\omega_{0}\left(-3D_{1n}+D_{2n}\right)}$	−9.5
−3	−1	$\omega_{c}-4\omega_{0}$	$k_{p}P_{-3}^{\mathrm{PM}}P_{-1}^{\mathrm{AM}}\cdot e^{-j\omega_{0}\left(-3D_{1n}-D_{2n}\right)}$	−9.5
1	7	$\omega_{c}+8\omega_{0}$	$k_{p}P_{1}^{\mathrm{PM}}P_{7}^{\mathrm{AM}}\cdot e^{-j\omega_{0}\left(D_{1n}+7D_{2n}\right)}$	−16.9
1	−7	$\omega_{c}-6\omega_{0}$	$k_{p}P_{1}^{\mathrm{PM}}P_{-7}^{\mathrm{AM}}\cdot e^{-j\omega_{0}\left(D_{1n}-7D_{2n}\right)}$	−16.9

**Table 2:** Characteristics of the dominant harmonic patterns of Equation (6) in decreasing order of significance for the case of 4-level AM and 8-level PM time-modulating waveforms. The useful harmonics are shaded in light blue.

*q*	*i*	Frequency	Dynamic Excitations (${}_{i}^{q}I_{n}$)	${}_{i}^{q}\Delta_{\mathrm{Peak}}$ (dB)
1	1	$\omega_{c}+2\omega_{0}$	$k_{p}P_{1}^{\mathrm{PM}}P_{1}^{\mathrm{AM}}\cdot e^{-j\omega_{0}\left(D_{1n}+D_{2n}\right)}$	0
1	−1	$\omega_{c}$	$k_{p}P_{1}^{\mathrm{PM}}P_{-1}^{\mathrm{AM}}\cdot e^{-j\omega_{0}\left(D_{1n}-D_{2n}\right)}$	0
1	7	$\omega_{c}+8\omega_{0}$	$k_{p}P_{1}^{\mathrm{PM}}P_{7}^{\mathrm{AM}}\cdot e^{-j\omega_{0}\left(D_{1n}+7D_{2n}\right)}$	−16.9
1	−7	$\omega_{c}-6\omega_{0}$	$k_{p}P_{1}^{\mathrm{PM}}P_{-7}^{\mathrm{AM}}\cdot e^{-j\omega_{0}\left(D_{1n}-7D_{2n}\right)}$	−16.9
−7	1	$\omega_{c}-6\omega_{0}$	$k_{p}P_{-7}^{\mathrm{PM}}P_{1}^{\mathrm{AM}}\cdot e^{-j\omega_{0}\left(-7D_{1n}+D_{2n}\right)}$	−16.9
−7	−1	$\omega_{c}-8\omega_{0}$	$k_{p}P_{-7}^{\mathrm{PM}}P_{-1}^{\mathrm{AM}}\cdot e^{-j\omega_{0}\left(-7D_{1n}-D_{2n}\right)}$	−16.9

**Table 4:** Performance comparison –in terms of power efficiency η, phase sensitivity $\theta_{\mathrm{step}}$, hardware complexity, and cost effectiveness– between the proposed dual-beam steerable TMA approach controlled with combined AM and PM waveforms (shaded in blue) and competitive solutions existing in the literature.

Architecture	η(dB)	$\theta_{\mathrm{step}}{(}^{\circ })$	SPDT Switches	Splitters	TMA Cost Savings (%)
standard PA	5.64	5.6	24	2	-
TMA AM [18]	5.67	1.7	9	8	114.2
TMA AM/PM [this work]	4.94	1.7	7	2	245.2

## Data Availability

Not applicable.

## Abbreviations

The following abbreviations are used in this manuscript:

AF	array factor
AM	amplitude modulation
BS	beamsteering
BFN	beamforming network
DOA	direction of arrival
DSB	double sideband
PM	phase modulation
RF	radio frequency
SP4T	single-pole four-throw
SP8T	single-pole eight-throw
SPDT	single-pole dual-throw
SSB	single sideband
TMA	time-modulated array
VPS	variable phase shifter
ULA	uniform linear array
WSN	wireless sensor network
